# Optimal treatment for penile verrucous carcinoma: a systematic literature review

**DOI:** 10.1186/s12894-020-00777-1

**Published:** 2021-01-29

**Authors:** Dong In Jo, Song Hyun Han, Soon Heum Kim, Hye Young Kim, Hong Chung, Hong Sup Kim

**Affiliations:** 1 Department of Plastic and Reconstructive Surgery, Konkuk University School of Medicine, Chungju, Republic of Korea; 2 Department of Anesthesiology and Pain Medicine, Konkuk University School of Medicine, Chungju, Republic of Korea; 3 Department of Urology, Konkuk University School of Medicine, Gukwon-daero 82, 27376 Chungju, Chungbuk Republic of Korea

**Keywords:** Local excision, Penile verrucous carcinoma, Squamous cell carcinoma

## Abstract

Verrucous carcinoma, a rare low-grade well-differentiated squamous cell carcinoma, is known for its favorable biological behavior and lack of metastatic potential. However, aggressive resection is problematic in terms of compromised function and aesthetics. Hence, more conservative treatments are needed. Methods: To identify the up-to-date general biological behavior, diagnosis, and treatment trends, we searched PubMed using the keyword “penile verrucous carcinoma” without restrictions on publication date. Results: Current treatments for penile verrucous carcinoma include wide surgical excision, seldom preventive lymphadenectomy, and conservative chemotherapy without surgery or local excision with safe margins. Despite the advent of partial penectomy to minimally impact function and aesthetics, affected patients experience psychosexual problems. Local excision can be used to save the penile shaft and glans penis without preventive lymphadenectomy or adjuvant therapy and can achieve good clinical prognosis with rare recurrence. Conclusions: To preserve the functional and cosmetic aspects, we recommend local excision, especially for tumors measuring < 3 cm and classified as stage T1 according to the 2016 tumor node metastasis clinical and pathological classification for penile cancer.

## Background

Verrucous carcinoma, a rare low-grade well-differentiated squamous cell carcinoma (SCC), is known for its slowly compressive expanding warty growth and rare metastasis [1–3]. Aggressive treatment, such as penectomy, has been suggested based on the concept that penile verruca carcinoma (PVC) is malignant. In contrast, less aggressive treatment, such as local excision without preventive lymphadenectomy, has been suggested based on the concept that the biological behavior of PVC resembles that of a benign tumor. In patients who had undergone aggressive wide surgical excision of the glans penis and penile shaft, many functional, cosmetic, and psychosexual problems have been reported. To date, surgical treatment trends have been unclear regarding the use of preservation surgery. Here we reviewed the literature to determine the most effective treatment for PVC and suggest best practices for treatment guidelines.

## Methods

To summarize the prevalence, causative factors, diagnostic methods, treatment methods, clinical behaviors, diagnostic imaging techniques, and prognosis of PVC, we searched PubMed for relevant studies using the keyword “penile verrucous carcinoma” without restrictions on publication year and retrieved abstracts published in English that mentioned PVC diagnosis and treatment. The abstracts were screened according to inclusion and exclusion criteria. The inclusion criteria were presented cases and the corresponding treatment methods, microscopic diagnosis of PVC, availability of full text articles, and publication in English. The exclusion criteria were lack of tumor staging information and tumor staging beyond T2 (Stage T2 penile cancers are different from penile verrucous carcinoma and defined as invasive cancers such as squamous cell carcinoma and others). To check the association between treatment method and conditions of cases, the treatments were categorized into 2 groups—the less aggressive treatment group included shaving, local excision, and no surgery and the aggressive treatment group included glansectomy, partial penectomy, and total penectomy. Individual factors included age, case history, tumor size, tumor shape, tumor location, tumor stage, adjuvant treatment, lymph node metastasis, disease-free status, recurrence, human papilloma virus (HPV) infection status, and treatment trends over time.

The statistical analysis was performed using SPSS for Windows version 25 (SPSS Inc., Chicago, IL, USA). We hypothesized that aggressive treatment is more effective and investigated the difference in efficacy between the 2 treatments. We performed a t-test, a chi-squared test, and Fisher’s exact test to ensure data accuracy and a regression analysis to examine whether the individual factors were correlated in the 2 groups.

## Results

**Fig. 1:**
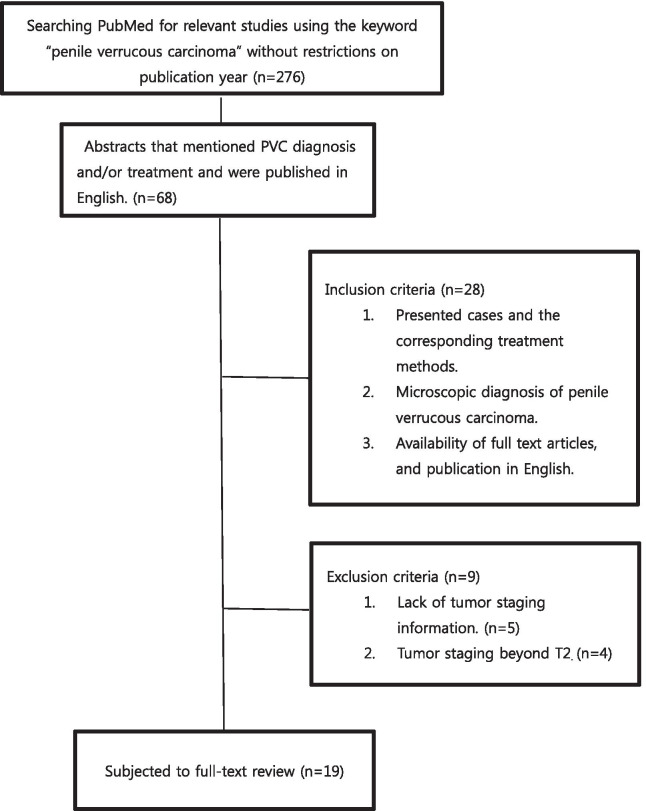
The study flow chart

A total of 276 articles were retrieved from PubMed. Among them, 68 abstracts that mentioned PVC diagnosis and/or treatment and were published in English were selected. The studies were published between 1969 and February 2019. Most of the studies were published in English; other publication languages included Spanish (14), Chinese (3), French (3), Japanese (2), Bulgarian (1), Israeli (1), and Italian (1). Several studies mentioned regarding PVC treatment and were simple case reports [4–47]. Its rarity is supported by the fact that 1 case in a 10-year period and 13 cases in a 30-year period were reported [10, 48–51]. Among the 68 studies retrieved, 28 met the inclusion criteria. Of those, 9 were excluded according to the exclusion criteria. Thus, a total of 19 studies were subjected to full-text review (Fig. 1) [4–7, 10, 11, 20, 23, 31, 32, 35, 37–40, 43, 45–47].

The studies comprised a total of 58 cases of PVC (Table 1). The patients were 28–86 years of age. The case histories were 1–204 months long. The tumor sizes were 0.8–10 cm. Fifty-4 of the cases included a description of tumor shape: papillary cauliflower in 30, keratotic hornlike in 3, and warty verrucous in 21. The tumors involved the glans in 33 cases, coronoid sulcus in 4, shaft in 4, prepuce in 7, prepuce and glans in 4, glans and coronoid sulcus and shaft in 1, prepuce and coronoid sulcus in 2, glans and coronoid sulcus in 2, and coronoid sulcus and shaft in 1. A total of 10 cases involved the coronoid sulcus, an area in which penile carcinoma would more rapidly infiltrate the penile fascia. Regarding staging, 52 cases were classified as stage Ta, 4 were classified as T1, and 2 were classified as T1a. Regarding treatment, no surgery was performed in 4 cases, local excision was performed in 10, shaving in 3, Mohs surgery was performed in 2, circumcision was performed in 1, glansectomy was performed in 3, partial penectomy was performed in 29, and total penectomy was performed in 6. Thus, 20 cases involved less aggressive treatment and 38 cases involved aggressive treatment. Fifteen cases of adjuvant treatment were reported, including 1 of radiotherapy, 6 of chemotherapy, 2 of chemoradiotherapy, and 6 of local therapy (CO_2_ laser, cryotherapy, intralesional interferon, and topical fluorouracil). There was only 1 case of lymph node metastasis. The reported lymphadenopathies were revealed as inflammation without metastasis [4–6]. One case of bilateral lymph node metastasis, which was suspected as a mixed tumor condition with moderately differentiated SCC, was reported [35]. The follow-up period was 6–228 months (19 years). There were 7 cases of tumor recurrence: 5 in the less aggressive treatment group and 2 in the aggressive treatment group. All cases except 2 achieved tumor-free status. The other 2 patients died due to other malignant conditions [35, 47]. Six cases were associated with the following diseases: anaplastic spindle cell carcinoma suggestive of malignant transformation after radiotherapy, hybrid verrucous SCC, moderately differentiated SCC, lichen sclerosis, pseudoepitheliomatous keratotic and micaceous balanitis, and human immunodeficiency virus infection. HPV infection status was described as negative in 8 cases; the others were not specified. The reported cases following the year of issue categorized in 10-year increments: 1 in the 1970s, 14 in the 1980s, 2 in the 1990s, 15 in the 2000s, and 26 in the 2010s. The main diagnostic method was biopsy. Chest x-ray, HPV polymerase chain reaction (PCR), ultrasonography, and computed tomography were optional. The best diagnostic method was biopsy and HPV PCR. Ultrasonography, computed tomography, and magnetic resonance imaging (MRI) offered more precise information about tumor anatomy and regional lymphadenopathy. Two reports mentioned surgical treatment guidelines according to tumor size [8] and depth [17]. Concerning surgical excision, the main treatment was radical surgery, including at least partial penectomy [4–47], even with the relatively small tumor size (< 3 cm) [8]. The surgical margins were 2 cm in cases of partial penectomy and 0.3–1 cm in cases of local excision (Table 2) [5, 6]. In contrast, some studies have emphasized the good clinical results of local excision because of the favorable clinical behavior of PVC [5, 6, 11, 15, 17, 31, 32, 37, 39, 40, 43, 45–47].

**Table 1:** Clinical manifestations of penile verrucous carcinoma

Case (Ref.)	Age (years)	Case history (months)	Tumor size (Cm)	Tumor shape	Tumor location	Stage (TNM)	Operation	Adjuvant threatment	Lymphnode metasatsis	Follow up (months) /disease free	Recurrence (months after)	Associated disease	HPV	Year of issue
1(47)	49	12	5 × 4.5 x 2	Papillary cauliflower	Glans, coronoid sulcus,	Ta	local excision	Systemic bleomycine, methotrexate,	none	48/no	24	Anaplastic spindle cell carcinoma	Negative	1994
					shaft			Radiotherapy				Malignant transformation after Radiotherapy		
2(46)	62	3	3	Cauliflower-like	Prepuce, coronal sulcus	Ta	Local excision	None	None	54/yes	26	Hybrid verrucous-squamous cell carcinoma	Negative	2000
3(45)	42	4	7	Cauliflower-like	Prepuce, glans	Ta	Local excision	CO_2_ laser	None	36/yes	None	None	?	2008
4(43)	51	24	?	Verrucous	Glans, coronary sulcus	Ta	Recurred mass	Liquid nitrogne, topical 5% fluorouracil	None	42/yes	None	None	?	1978
							after local excision							
							and skin graft							
							after local excision							
5(40)	60	?	?	?	Glans	Ta	Shaving	Intralesional interferone	None	30/yes	None	None	Negative	2000
6(39)	69	24	1	Keratotic	Glans	Ta	Shaving	Cryosurgery with liquid nitrogen	None	36/yes	None	None	?	2002
7(39)	69	24	1.5	Keratotic	Glans	Ta	Shaving	Cryosurgery with liquid nitrogen	None	36/yes	None	None	?	
8(38)	27	12	5 × 5	Verrucous	gLans	Ta	None	Intra-aortic infusion with methotrexate	None	214/yes	None	None	?	2003
9(38)	65	3	4 × 3	Warty	Glans	Ta	None	Intra-aortic infusion with methotrexate	None	165/yes	None	None	?	
10(38)	31	48	5 × 5	?	Shaft	Ta	Total penectomy	Intra-aortic infusion with methotrexate	None	149/yes	36/partial	None	?	
							due to partial response				Response			
							and unbarable pain							
11(38)	75	120	2 × 2	?	Glans	Ta	None	Intra-aortic infusion with methotrexate	none	104/yes	None	None	?	
12(37)	70	?	?	Verrucous	Shaft, base of penis	T1	Mohs surgery and FTSG	Cisplatin and fluorouracil with radiotherapy	none	36/yes	16	None	negative	2009
							after recurrence (local excision)							
13(35)	47	3	4 × 3	?	Prepuce, glans	Ta	Total penectomy	Intra-aortic infusion with methotrexate	Bilateral	37/no	18/partial	Moderately differentiated squamous cell	?	2010
							due to partial response				Response	Carcinoma, malignant transformation?		
14(35)	28	72	5 × 4.5x2	Verrucous	Prepuce, glans	Ta	None	Intra-aortic infusion with methotrexate	None	45/yes	None	None	?	
15(32)	42	?	1	Warty	Glans	Ta	Mohs surgery	None (healed by secondary intention)	None	12/yes	None	Lichen sclerosus	?	1987
16(31)	74	12	2 × 1.5	Warty	Glans	Ta	Local excision	Failed cryotherapy with liquid nitrogen	None	48/yes	None	Pseudoepitheliomatous	negative	2000
				Nodule			(surgical margin 2 cm)					Keratotic and micaceous balanitis		
17(23)	60	7	3 × 3	Verrucous, ulcerative	Prepuce, glans	Ta	Partial penectomy	None	None	24/yes	None	Human immunodeficiency virus infection	negative	2015
18(20)	71	6	2.8 × 1.6	Keratotic hornlike	Glans	T1a	Partial penectomy	None	None	10/yes	None	None	Negative	1990
							total penectomy	(due to residual tumor on the resection margins)						
19(11)	61	?	7 × 4	Cauliflower-like	Shaft	T1a	Local excsion, skin graft	None	None	36/yes	None	None	?	2019
20(10)	30 to 86	at least 12	1 to 8	Warty or fungating	11 glans	10 Ta	9 partial penectomy	All none but	All	72 to 228	All	All none	All	1985
	mean 47	in 10 cases	mean 3.6	(multiple nodules	2 prepuce	3 T1	3 total penectomy	1 radiotherapy	none	/yes	none		?	
		(in 5 cases		in 7 cases)			1 circumcision	(before total penaectomy)						
	13 cases	2,2,4,8,17 years)												
		in 3 cases												
32(10)		(1,2,4 months)												
33(7)	40 to 63	8 to 25	2.5 to 6.2	Cauliflower-like	Glans	Ta	Glansectomy	None	None	18 to 65	None	None	?	2001
	mean 54 ± 7						with frozen section			mean 38 ± 14				
34(7)				Cauliflower-like	Glans	Ta	Glansectomy	None	None	/Yes	None	None	?	
35(7)				Cauliflower-like	Glans	Ta	Partial penectomy after	None	None		3	None	?	
							glansectomy							
36(7)				Cauliflower-like	Glans	Ta	Glansectomy	None	None		None	None	?	
37(6)	73	12	3	Verrucous	coronary sulcus, shaft	Ta	Local excision	None	None	24/yes	None	None	Negative	2017
38(5)	52	96	6	All	coronoid sulcus	all	Partial penectomy	All none	All	6 to 60	All	Squamous atypical hyperplasia	All	2011
39(5)	85	48	3	Exophytic papillary	Glans	Ta	Local excision		None	mean 36	None	None	?	
40(5)	55	24	5	Cauliflower-like	Coronoid sulcus		Partial penectomy			/yes		None		
41(5)	64	3	2		Glans		Partial penectomy					None		
42(5)	74	3	3		Glans		Local excision					Squamous papilloma		
43(5)	56	12	2		Glans		Local excsion					Squamous atypical hyperplasia		
44(5)	52	3	2.5		Coronoid sulcus		Partial penectomy					None		
45(5)	55	24	10		Shaft		Partial penectomy					Squamous atypical hyperplasia		
46(5)	68	60	2		Coronoid sulcus		Circumcision,	Surgical margin 2 cm for partial penectomy				None		
							Partial penectomy	0.5–1 cm for local excision						
47(5)	70	6	4.5		Glans		Partial penectomy	Acknowledging excessive resection in 3 cases				None		
48(5)	49	3	3		Glans		Partial penectomy	For the small-sized mass limited to glans				None		
49(4)	35 to 72	All ?	0.8 to 4	All	5 prepuce	All	All	All none	All	8 to 108	All	All	All	2015
50(4)	mean 51.5			Cauliflower-like	1 prepuce, coronoid sulcus	Ta	Partial penectomy		None	/Yes	but 1 case	but 1 squamous metaplasia with	?	
51(4)					1 glans, coronoid sulcus						36, 60, 84	partial hyperkeratosis		
52(4)					3 glans									
53(4)														
54(4)	10 cases													
55(4)														
56(4)														
57(4)								8 Biopsy before surgical treatment						
58(4)								2 circumcision before surgical treatment						

?; missing individual data, Ref.; reference number

**Table 2:** Cumulative data of clinical presentations and treatments

Total cases	58
Age (years)	28–86
Case History (months)	1–204
Size (Cm)	0.8–10
Tumor shape	
Papillary cauliflower Keratotic hornlike Warty verrucous Unknown	30 3 21 4
Location (cases)	
Glans Coronoid sulcus Shaft Prepuce Prepuce and glans Glans and coronoid sulcus and shaft Prepuce and coronoid sulcus Glans and coronoid sulcus Coronoid sulcus and shaft Coronoid sulcus involvement	33 4 4 7 4 1 2 2 1 10
Stage (cases)	
Ta T1	52 6
Treatment (cases)	
No surgery Local excision Shaving Mohs surgery Circumcision Glansectomy Partial penectomy Total penectomy	4 10 3 2 1 3 29 6
Adjuvant treatment (cases)	
Radiotherapy Chemotherapy Chemoradiotherapy Local therapy (CO_2_ laser, cryotherapy, intralesional interferon, and topical fluorouracil)	1 6 2 6
Lymphnode metastasis (cases)	1
Follow up period (months)	6–228
Recurrence (cases)	7
Disease free (cases)	56
Surgical margin (Cm)	0.3–2
Cases following the year of issue (cases)	
1970s 1980s 1990s 2000s 2010s	1 14 2 15 26

**Table 3:** Cross analysis (chi-square) with Fisher’s exact test

			Operation		Total	χ^2^ (p)
			Less aggressive treatment	Aggressive treatment		
Tumor location	Glans	Case	11	22	33	2.532 (.686)
		%	55.0%	57.9%	56.9%	
	Coronoid sulcus involvement	Case	4	6	10	
		%	20.0%	15.8%	17.2%	
Stage	Ta	Case	17	35	52	.713 (.405)
		%	85.0%	92.1%	89.7%	
	T1	Case	3	3	6	
		%	15.0%	7.9%	10.3%	
Adjuvant treatment	none	Case	8	35	43	21.926** (.000)
		%	40.0%	92.1%	74.1%	
	Radiotherapy	Case	0	1	1	
		%	0.0%	2.6%	1.7%	
	Chemotherapy	Case	4	2	6	
		%	20.0%	5.3%	10.3%	
	Chemoradiotherapy	Case	2	0	2	
		%	10.0%	0.0%	3.4%	
	Local therapy	Case	6	0	6	
		%	30.0%	0.0%	10.3%	
Recurrence	No	Case	17	34	51	.247 (.619)
		%	85.0%	89.5%	87.9%	
	Yes	Case	3	4	7	
		%	15.0%	10.5%	12.1%	
Treatment trends over time following the year of issue	1970s	Case	1	0	1	12.549** (.005)
		%	5.0%	0.0%	1.7%	
	1980s	Case	2	12	14	
		%	10.0%	31.6%	24.1%	
	1990s	Case	1	1	2	
		%	5.0%	2.6%	3.4%	
	2000s	Case	10	5	15	
		%	50.0%	13.2%	25.9%	
	2010s	Case	6	20	26	
		%	30.0%	52.6%	44.8%	

*p < 0.05, **p < 0.01

The results of the t-test using categories of age, case history, and tumor size and the result of the cross-sectional analysis of the categories of tumor shape, disease-free status, and HPV status were excluded since some individual data for each case were missing (data n ot shown). In the cross-sectional analysis, tumor location, tumor stage, and recurrence were not significantly associated with either treatment. Regarding the clinical results, all but 2 patients (who died of other malignant conditions) achieved disease-free status. Regarding treatment efficacy, the recurrence rates did not differ significantly between the less aggressive and aggressive treatments. Patients who received adjuvant therapy tended to ultimately receive less aggressive treatment. Regarding the test statistics, the X^2^ was 21.926 and the probability was 0.000. Thus, the results were statistically significant. Regarding differences in treatment trends over time, the X^2^ was 12.549 and the probability was 0.005. Thus, the result was statistically significant (Table 3).

According to the regression analysis of age, case history, tumor size, tumor shape, tumor location, tumor stage, adjuvant treatment, tumor recurrence, and treatment trends over time did not appear to have a significant negative or positive effect (data not shown).

In summary, the associations between tumor location and treatment method and tumor depth and treatment method were not statistically significant. Adjuvant therapy tended to be performed alone or with local excision preventing a penectomy or glansectomy. Partial penectomy cases (aggressive treatment group) were predominantly reported in the 2010s. No intergroup differences were seen in clinical results. Therefore, our hypothesis that aggressive treatment is more effective was rejected.

### Discussion

Some studies have reported that PVC is observed in approximately 2.4–24% of all penile cancers and 20% of verruciform lesions of the penis; PVC is also observed in patients with Buschke–Löwenstein, warty carcinoma, and papillary SCC [1–3]. Several cases have been reported during the past 2–3 decades among many countries due to its rarity [10, 48–51]. PVC primarily occurs in the glans penis, and phimosis and redundant prepuce are 2 of its important causes [2, 52]. Lichen sclerosus and pseudoepitheliomatous, keratotic, and micaceous balanitis are other possible causes [31–33, 53]. Local squamous epithelial hyperplasia and hyperkeratosis may be important in the development of PVC [54, 55]. Clinically, they do not cause significant pain, but they grow slowly and uninhibited, sometimes invading the shaft over the glans. In most cases, the patients present with a slow-growing mass with multiple papillary lesions [4–6].

Biopsy and HPV PCR tests are basic diagnostic tools for differentiating PVC from HPV-related tumors. Increased immunohistochemical expression of markers such as Mdm2 and Ki67 and low expression of Bcl-2 may be useful for the detection of PVC [56–58]. Microscopically, the hematoxylin- and eosin-stained sections shows extension of the epithelium downward into the underlying tissues in a bulbous or drumstick process, while the tumor exhibits clear boundaries and rich lymphocytic infiltration into the surrounding mesenchyme [4–6].

To avoid misdiagnosis, repeated deeper biopsies are recommended that include the basement membrane of the papillomatous tumor, especially in cases in which PVC is highly suspected. However, because the gross morphology of PVC is very similar to that of condyloma acuminatum, it can be difficult to identify. HPV is known to be closely associated with penile cancer and condyloma acuminatum in most cases [8]. In contrast, in all PVC cases, the pathogenesis is not associated with HPV infection [3, 59–61]. Thus, an HPV-negative status may be the key in the differential diagnosis of PVC. In our study, the differential diagnosis from condyloma acuminatum was confirmed in only 8 cases. We assume that diagnostic biopsy played a decisive role since HPV infection status was unknown.

Surgical treatments reported in other studies focused on aggressive treatments, including glansectomy and partial or total penectomy with a 4–20-mm surgical margin [4, 5, 7, 8]. Partial penectomy with a 2-cm margin has traditionally been the suggested treatment for tumors involving the glans penis, with total penectomy being indicated when the tumor involves a larger portion of the penile shaft [49].

However, since PVC has a relatively rare incidence and is termed carcinoma despite its favorable behavior, surgeons often lack of experience treating such cases and decide to unnecessarily remove part or all of the penis. Moreover, wide excision was commonly performed for relatively small masses (≤ 3 cm) [5]. However, since the 1980s, local excision has been advised to preserve the penis [4–6],15,17. Mohs surgery was adopted in cases of PVC showing favorable behavior [15, 62]. The authors agree that local excision should be the first choice of treatment because of the favorable biological behavior of PVC. Treatments have been suggested according to 2 general concepts: penectomy is mandatory because PVC is malignant; and less aggressive treatment as local excision is sufficient because the biological behavior of PVC resembles that of a benign tumor.

This review revealed that the glans was the area most often involved in cases of PVC. We expected that distal and local lesions would be treated less aggressively. However, as a result, tumor location did not affect treatment aggressiveness. Interestingly, the coronoid sulcus involvement suggests that, in the absence of a dartos layer, penile carcinoma would more rapidly infiltrate the penile fascia, a known low-resistance pathway for local spread; thus, clinicians would expect a higher risk of tumor recurrence and inguinal involvement as well as a worse outcome. Thus, we expected that coronoid sulcus involvement would require more aggressive treatment. However, our results demonstrated 11 cases in the less aggressive treatment group versus 22 cases in the aggressive treatment group, respectively. Although there were more cases in the latter than the former group, the intergroup difference was insignificant.

Regarding tumor depth, PVC is defined as a superficial stage Ta lesion by the 2016 Tumor Node Metastasis (TNM) classification, a so-called non-invasive verrucous carcinoma. Although no statistically significant intergroup difference was noted, aggressive treatments were more often applied than less aggressive treatments for superficial lesions. However, 20 cases of the less aggressive treatment group showed good clinical results. Stage T1 tumors were seen, even in cases of deeper lesions. This means that less aggressive treatments with careful follow-up of stage T1 tumors can also result in good post-treatment results.

Even if a case of PVC is malignant, it may present as a benign tumor. Thus, to preserve functional and cosmetic results, we recommend that local excision with minimal surgical margins followed by careful observation be the first-line choice of treatment, especially for tumors measuring < 3 cm and classified as stage T1. In other conditions, the tumor should be considered not PVC and the excision should be widened. In our study, we excluded tumor staging beyond T2. Stage T2 penile cancers are different from PVC and defined as invasive cancers such as squamous cell carcinoma and others with bad prognosis. In these cases, aggressive treatment is recommended.

Regarding adjuvant therapy, preventive inguinal lymphadenectomy was hardly used because of rarity of evident lesions [4, 5, 9, 34, 35, 63, 64]. Conservative systemic chemotherapy without surgery was reported [35, 38]. Other adjuvant therapies for the verrucous lesion have been introduced, such as topical aminolevulinic acid–photodynamic therapy; topical, systemic, or intralesional interferon; cryotherapy; laser therapy; and radiation [35–45, 65, 66]. Our results demonstrated that adjuvant treatments were more predominantly applied when less aggressive treatment was administered. This finding supports that conservative surgery could be the first choice of treatment. However, the 4 cases treated with intralesional interferon and 1 case of cryotherapy with good clinical results could not be evaluated due to the absence of information on tumor stage [25, 41, 42].

This literature review revealed that inguinal lymphadenectomy was performed in certain patients; however, no evident lesions were found in such cases [4, 5, 9, 34, 35, 63, 64]. The 1 case of lymph node metastasis reported was suspected to be a combined lesion with moderately differentiated SCC [35]. Thus, we agree that inguinal lymphadenectomy is not an appropriate prophylactic treatment. For lymphadenopathy, treatment with anti-inflammatory drugs may be the treatment of choice, followed by a lymph node biopsy as needed. Thus, if a case of PVC is confirmed by biopsy and no signs of inguinal lymphadenopathy are seen on physical examination, further workups such as computed tomography or ultrasonography could be postponed initially, and high-end MRI saved for later and then used if needed to investigate tumor depth [67].

As for tumor behavior, complicated microlesions of invasive SCC, a certain number of which eventually progressed to other invasive types, have been observed in < 30% of the reported cases of PVC [46, 68]. There was one case of recurrent SCC after anaplastic transformation following radiation therapy [47]. Therefore, close follow-up for the early detection of any sign of recurrence requiring additional resection is essential after a less aggressive treatment, such as local excision. In our study, all but 2 cases achieved tumor-free status during long follow-up periods despite 7 cases of recurrence. Regarding those 2 cases, 1 was suspected as malignant transformation after radiotherapy to anaplastic spindle cell carcinoma [47] and the other was the previously mentioned lymph node metastasis case that eventually failed treatment and required total penectomy due to partial response after chemotherapy [35].

Despite the favorable clinical behavior of PVC and the many studies emphasizing less aggressive treatments, the use of aggressive treatment was predominantly reported in the 2010s. However, we do not think that this reflects the recent treatment trends because the timing of the reported treatment does not represent the actual clinical practice at the time.

However, information is still lacking about the association between treatment and tumor condition, evidence of which could lead us to define an appropriate guideline. Due to the limitations of a literature review, controllable factors were often undetermined. Thus, we recommend that future studies always include a unified scale for multiple factors including tumor condition and functional outcome. This mission will require long discussions and consensus of many experts. Despite this limitation, we believe that our findings are meaningful since this is the first review of diagnostic and treatment trends of PVC, a rare condition.

## Conclusion

The review performed here revealed that PVC tends not to recur or metastasize after resection but that surgical treatment tends to remove too much tissue. However, in most cases of local excision, the wound heals well and local recurrence rarely occurs. Therefore, considering the ability of local excision with minimal surgical margins to spare the functional and cosmetic aspects of the penile shaft and glans penis, we recommend it as the first-line choice of treatment with observation, especially for tumors measuring < 3 cm and classified as stage T1 according to the 2016 tumor node metastasis clinical and pathological classification for penile cancer.

## Data Availability

Not applicable.

## Abbreviations

HPV: Human papilloma virus
MRI: Magnetic resonance imaging
PCR: Polymerase chain reaction
PVC: Penile verruca carcinoma
SCC: Squamous cell carcinoma
TNM: Tumor node metastasis
